# Elevated Blood S100B Levels in Patients With Migraine: A Systematic Review and Meta-Analysis

**DOI:** 10.3389/fneur.2022.914051

**Published:** 2022-07-14

**Authors:** Chaojia Chu, Rui Zhong, Mengtan Cai, Nan Li, Weihong Lin

**Affiliations:** Department of Neurology, Neuroscience Center, The First Hospital of Jilin University, Changchun, China

**Keywords:** S100B, blood levels, migraine, meta-analysis, case-control

## Abstract

**Background:**

In recent years, a growing number of researches indicate that S100B may act in migraine, but the relationship between S100B and migraine remains controversial. Therefore, the current study aimed to perform a meta-analysis to quantitatively summarize S100B levels in migraine patients.

**Methods:**

We used Stata 12.0 software to summarize eligible studies from PubMed, EMBASE, Web of Science, Cochrane Library, China National Knowledge Infrastructure (CNKI), and Wanfang databases. We applied standardized mean differences (SMDs) with 95% confidence intervals (95%CIs) to appraise the association between S100B and migraine.

**Results:**

The combined results of nine case-control studies indicated that compared with healthy controls, overall migraine patients had significantly increased S100B levels in peripheral blood (SMD = 0.688, 95%CI: 0.341–1.036, *P* < 0.001). The S100B levels in migraineurs during ictal periods (SMD =1.123, 95%CI: 0.409–1.836, *P* = 0.002) and interictal periods (SMD = 0.487, 95%CI: 0313–0.661, *P* < 0.001), aura (SMD = 0.999, 95%CI: 0.598–1.400, *P* < 0.001) and without aura (SMD = 0.534, 95%CI: 0.286–0.783, *P* < 0.001) were significantly higher than those in the controls. The subgroup analyses by age, country, migraine assessment, and assay method of S100B also illustrated a statistically obvious association between S100B levels and migraine, indicating that age may be the most important source of heterogeneity. Sensitivity analysis showed that no individual study has a significant influence on the overall association between S100B and migraine.

**Conclusion:**

This meta-analysis demonstrates that the level of S100B in peripheral blood of patients with migraine was significantly increased. Migraine may be associated with pathological reactions involving S100B, which is instrumental for the clinical diagnosis of migraine and therapy that considers S100B as a potential target.

## Introduction

Migraine has been known as a leading cause of disability worldwide through the ongoing Global Burden of Diseases, Injuries, and Risk Factors Study (1, 2), with an estimated prevalence between 2.6 and 21.7% in different regions and populations (3). The prevalence of migraine in women is higher than that in men, and the ratio of male to female prevalence changes with age (4). The severe attack and chronic course of migraine can affect the patient's academic performance, occupation, psychological health, quality of life, social interaction, and even financial stability of individual family members (5–7) which is an important component increasing the burden of public health. Migraine is very prevalent, but its pathogenesis remains unclear. Cortical spreading depression (8), trigeminal system (9), vasodilation (10), and hypothalamus (11, 12) may be involved in the triggering and occurrence of migraine. Increased synaptic connectivity and excitability, and expansion of receptive fields, that is, central sensitization, are relevant to the development of chronic migraine (13).

The S100 protein family comprises 24 calcium-binding proteins that exert intracellular or extracellular regulatory effects. They act as calcium-activated switches and regulate the activity of various targets in different tissues. S100B is mainly serried in astrocytes (14) and other glial cell types, such as oligodendrocytes and Schwann cells, and is not limited to the nervous system (15). S100B is involved in cell proliferation, survival, and differentiation (16). Originally, S100B was considered a marker of neural injury because it can leak from damaged cells. Currently, S100B was also found to be initiatively released by different cell types, specifically under stress status. It is directly related to the progress of a disease, including congenital/perinatal disorders, neurodegenerative diseases, acute brain injury, psychiatric disorders, and inflammatory bowel disease (16). S100B at high concentrations induced toxic/proinflammatory effects (17, 18).

The mechanism of migraine is not fully understood, and the release of many inflammatory factors involves the entire process and its chronicity. Calcitonin gene-related peptide (CGRP) levels increase during ictal periods of migraine, and experiments have shown that it originates from stimulating trigeminal nerve fibers (13), leading to the production and release of inflammatory cytokines and induction to sensitization of trigeminal neurons. Studies have found that chemical stimulation to the trigeminal nerves increases the expression of the inflammatory protein S100B in both neurons and glia. Increased S100B is thought to participate in peripheral sensitization of trigeminal neurons and takes a part in the formation and continuance of inflammatory pain (19). Tetrandrine has been reported to suppress the overexpression of S100B while significantly alleviating trigeminal pain sensitization (20). S100B may play an important pathological role in the occurrence of migraine and may be a potential target for the treatment of migraine. However, there are few studies on the relationship between S100B and migraine, and this relationship is unclear. Some studies believe that S100B rises during ictal or interictal, and some studies have shown that it will increase in the chronic phase. Therefore, this study performed a meta-analysis on the controversy of the relationship between the two in previous studies and explored the relationship between S100B and migraine.

## Materials and Methods

This meta-analysis was conducted in terms of the guidelines of the Preferred Reporting Items for Systematic Reviews and Meta-Analyses (PRISMA) (21).

### Search Strategies

A comprehensive search was executed in the PubMed, Web of Science, EMBASE, Cochrane Library, China National Knowledge Infrastructure (CNKI), and Wanfang databases by two independent investigators (CC and RZ) from inception to October 2021, with no restrictions on population. Languages are limited to English and Chinese. We used the following combination of subject headings and free terms: (S100 calcium binding protein beta subunit OR S100β OR S100B OR S100beta OR S100 calcium-binding protein B) and (migraine disorders OR migraine OR hemicrania OR headache). Detailed search term strategies are presented in the Supplementary Materials. We search the Chinese database for the following phrases: (S100B OR钙结合蛋白B OR 钙结合蛋白β) and (偏头痛) (“钙结合蛋白” means “calcium binding protein,” “偏头痛” means “migraine”).

### Study Selection

**Figure 1:**
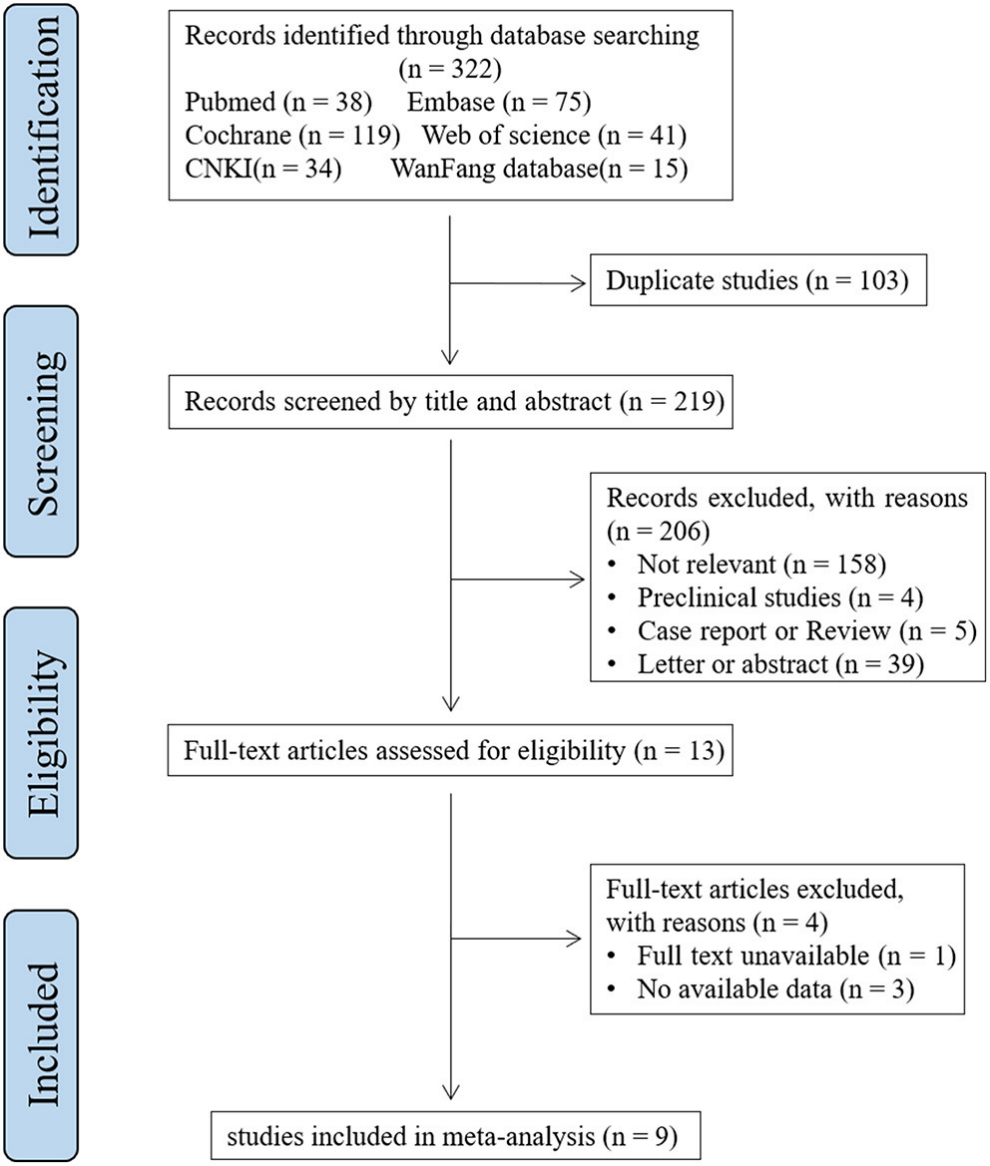
PRISMA flowchart for the literature search.

All potential studies were reviewed step-by-step by two researchers (CC and RZ) independently (Figure 1). Irrelevant studies on account of titles or abstracts were excluded first. Provisionally eligible studies were needed to be evaluated for eligibility by inspecting the full text. Any disagreements between the two reviewers were resolved by a third reviewer (MC). The studies included in this meta-analysis met the following inclusion criteria: a) Population: individuals diagnosed with migraine (according to international guidelines); b) Intervention (i.e. assessment): measurement of S100B reported in mean ± SD or can be converted to mean ± SD; c) Comparator: individuals without migraine; d) Outcome: the level of S100B; and e) Study designs: cross-sectional or case-control study. The exclusion criteria were as follows: a) conference abstracts, letters, case reports, reviews, and meta-analyze; b) no full text. Duplicated studies were removed. We included the latest or most complete study, for studies with reduplicated populations.

### Data Extraction

Information was extracted on the first author, publication year, country, age, gender distribution (female %), sample size, diagnosis, and study design (Table 1). When the original study only mentioned the migraine diagnostic criteria proposed by the International Headache Society (IHS), and did not specify which version of the criteria, was expressed by IHS. Data on mean S100B concentrations (mean ± SD) were extracted as primary outcomes and used for combining data by two independent researchers (Table 2).

**Table 1:** Characteristics of included studies.

	**Country**	**Age(mean/range)**		**Female (%)**		**Number**		**Migraine assessment**	**Study design**	**NOS score**
		**Case**	**Control**	**Case**	**Control**	**Case**	**Control**			
Gönen et al. (22)	Turkey	36.90	36.30	84.90	74.10	53	27	ICHD-III	Case-control	8
Riesco et al. (24)	Spain	40.50	39.50	88.70	100.00	62	29	ICHD-III	Case-control	7
Yilmaz et al. (29)	Turkey	33.88	36.40	67.30	56.70	52	30	ICHD-II	Case-control	7
Snoer et al. (25)	Denmark	48.00	42.00	81.30	66.70	32	6	ICHD-III	Case-control	8
Tian et al. (27)	China	30.98	30.03	49.10	52.00	53	55	ICHD-III	Case-control	8
Yilmaz et al. (28)	Turkey	36.41	33.97	90.20	74.30	41	35	IHS	Case-control	7
Teepker et al. (26)	Germany	19.00–56.00	Match^*^	61.90	Match^*^	21	21	IHS	Case-control	8
Zhang et al. (30)	China	34.32	Match^*^	70.00	Match^*^	40	38	IHS	Case-control	8
Papandreou et al. (23)	Greece	10.60	Match^*^	60.00	NR	15	23	IHS	Case-control	6

* *Match is mentioned in studies, but the detailed number is not reported. NR, not reported; ICHD-III, International Classification of Headache Disorders, 3rd edition; ICHD-II, International Classification of Headache Disorders 2nd edition; IHS, criteria of the International Headache Society*.

**Table 2:** Comparison of S100B levels.

	**S100B level (mean** **±SD, pg/ml)**						**Assay methods**	**The time for obtaining the samples**
	**Migraine**					**Control**		
	**Total**	**Ictal period**	**Interictal period**	**Patients with aura**	**Patients without aura**			
Gönen et al. (22)	133.29 ± 83.07	NR	133.29 ± 83.07	NR	133.29 ± 83.07	75.20 ± 36.10	ELISA	Interictal: no headache within 3 days
Riesco et al. (24)	22.38 ± 9.61	NR	22.38 ± 9.61	NR	NR	20.60 ± 8.30	ELISA	Interictal: migraine-free period
Yilmaz et al. (29)	81.90 ± 54.00	99.80 ± 73.00	71.10 ± 38.00	85.10 ± 59.00	79.00 ± 52.00	48.60 ± 12.00	ECLIA	Ictal: 2–5 hours after the onset Interictal: headache-free period
Snoer et al. (25)	40.00 ± 20.00	NR	40.00 ± 20.00	NR	NR	40.00 ± 20.00	ECLIA	Interictal: no headache within 2 days
Tian et al. (27)	35.91 ± 8.38	NR	35.91 ± 8.38	36.86 ± 7.04	35.33 ± 9.15	31.08 ± 4.78	ELISA	Interictal: no headache within 3 days
Yilmaz et al. (28)	124.00 ± 221.77	126.00 ± 215.00	122.00 ± 231.00	NR	124.00 ± 221.80	45.00 ± 21.00	ECLIA	Ictal: during attacks Interictal: no headache within 2 days
Teepker et al. (26)	61.00 ± 31.76	52.00 ± 23.00	70.00 ± 37.00	NR	NR	32.00 ± 160.00	other	Ictal: within 2 hours after onset Interictal: 2-4 days after the cessation
Zhang et al. (30)	0.71 ± 0.70	1.01 ± 0.81	0.41 ± 0.39	NR	NR	0.33 ± 0.21	ELISA	Ictal: during attacks or within 24 hours after cessation, Interictal: no headache within 7 days
Papandreou et al. (23)	210.00 ± 50.00	210.0 ± 50.0	NR	NR	NR	100.00 ± 20.00	other	Ictal: during or within 3 hours after attacks

*NR, not reported; ELISA, enzyme-linked immunosorbent assay; ECLIA, electrochemical luminescence immunoassay*.

### Quality Assessment

The quality of the included study was estimated by using the Newcastle-Ottawa Scale. This score classifies individual studies across three domains: selection, comparability, and exposure. Two researchers independently estimated the quality of the studies and resolved any disagreements through discussion.

### Statistical Analyses

Associations between S100B levels and migraine was assessed by using SMDs with 95%CIs describing continuous data, and the results are visualized as forest plots, which included the contribution of each study (weight) to the overall effect. In the present meta-analysis, results with two-tailed *P*-values < 0.05, were regarded as statistically significant. Substantial heterogeneity of the included studies was confirmed by >50% and *P*-value of Q test < 0.10). A random-effects model or a fixed-effects model was selected by *I*^2^-value. Sensitivity analyses were performed to examine the potential source of heterogeneity using leave-one-out cross-validation. Begg's rank correlation test and Egger's regression asymmetry test were employed to evaluate publication bias if the number of included studies was >8 in the meta-analysis. Stata (version 12.0, Stata Corp LP, College Station, Texas, USA) was used for the statistical analysis.

## Results

### Literature Search

The systematic literature search produced 322 references. In total, 103 duplicate references were removed first. We identified 13 studies that were potentially eligible for inclusion after scanning the titles and abstracts. After the full-text assessment, nine studies were evaluated for eligibility and included in the quantitative analysis (22–30). A flowchart of the process used to select the studies is shown in Figure 1.

### Characteristics and Quality Assessments of Included Studies

Nine included studies comprising 633 individuals (369 migraine cases and 264 controls) were published between 2005 and 2021. Two of the studies were conducted in China and seven in Europe.

The Newcastle-Ottawa Scale scores of the studies varied from 6 to 8 points (mean score: 6.81), implying the good quality of the included studies. Specific quality scores of the included studies are listed in Table 3.

**Table 3:** Study quality assessment based on Newcastle-Ottawa Scale (*n* = 9).

	**Selection**				**Comparability**	**Exposure**		
	**Is the case**	**Representativeness**	**Selection**	**Definition**	**Comparability of cases**	**Assessment**	**The same method of**	**Non-response**
	**definition**	**of the**	**of controls**	**of controls**	**and controls based on**	**of exposure**	**ascertainment for**	**rate**
	**adequate?**	**cases**			**the design or analysis**		**cases and controls**	
Gönen et al. (22)	1	1	1	1	2	1	1	0
Riesco et al. (24)	1	1	0	1	2	1	1	0
Yilmaz et al. (29)	1	1	0	1	2	1	1	0
Snoer et al. (25)	1	1	1	1	2	1	1	0
Tian et al. (27)	1	1	1	1	2	1	1	0
Yilmaz et al. (28)	1	1	0	1	2	1	1	0
Teepker et al. (26)	1	1	1	1	2	1	1	0
Zhang et al. (30)	1	1	1	1	2	1	1	0
Papandreou et al. (23)	1	1	0	1	1	1	1	0

### Results of Meta-Analysis

#### The Association of S100B Levels With Overall Migraine

**Figure 2:**
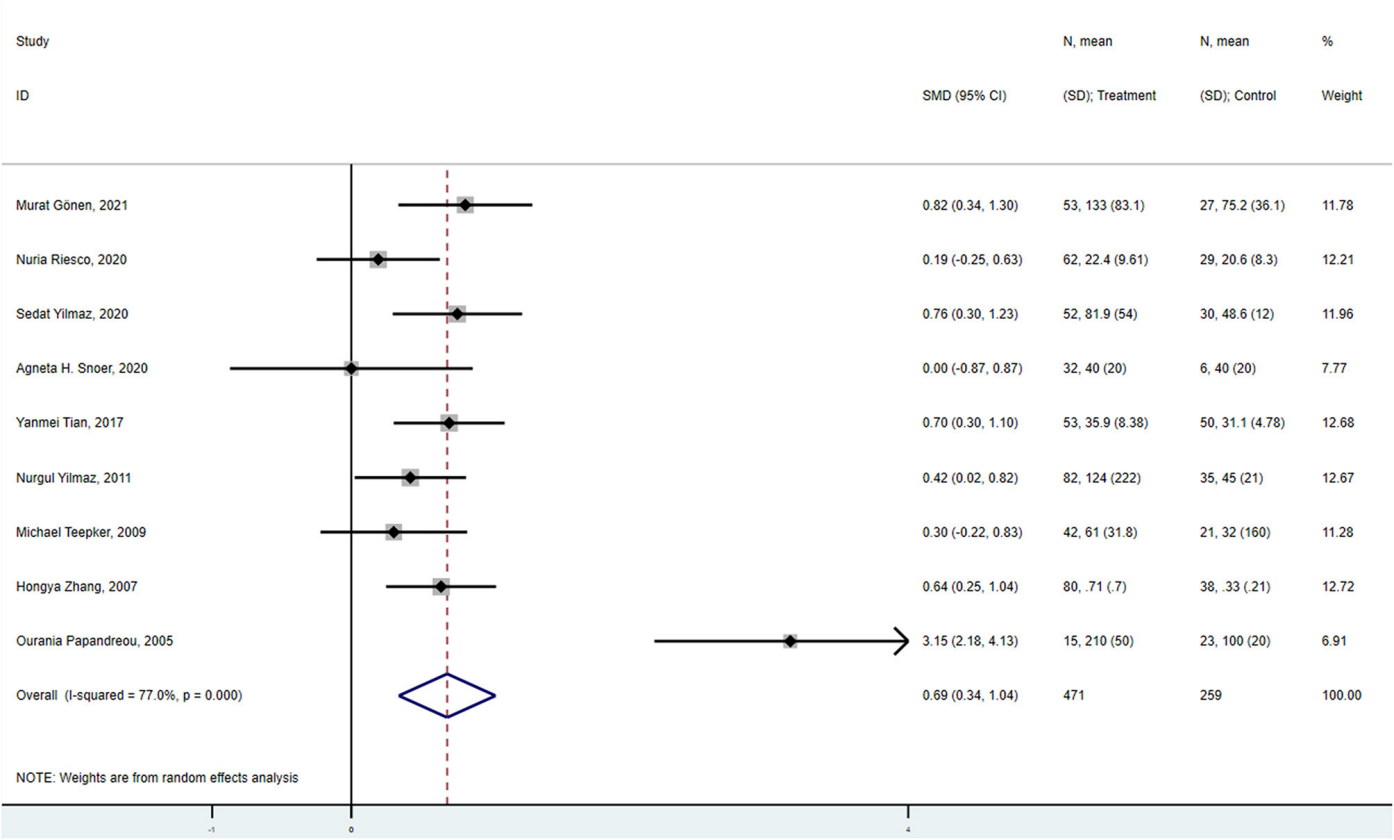
Forest plot for the association between S100B levels and overall migraine. SMD, standardized mean difference; CI, confidence interval.

A meta-analysis was carried out using a random-effects model due to the high heterogeneity (*I*^2^ = 77.0%, *P* < 0.001). Synthetic results illustrated that overall migraine patients (with or without aura, ictal or interictal) had significantly elevated S100B levels in peripheral blood compared to healthy controls (SMD = 0.688, 95%CI: 0.341–1.036, *P* < 0.001) (Figure 2).

#### S100B Levels of Migraineurs in Interictal and Ictal Periods

Of the nine studies included in the meta-analysis, eight studies contained S100B levels of migraineurs in the interictal period and five studies in the ictal period.

**Figure 3:**
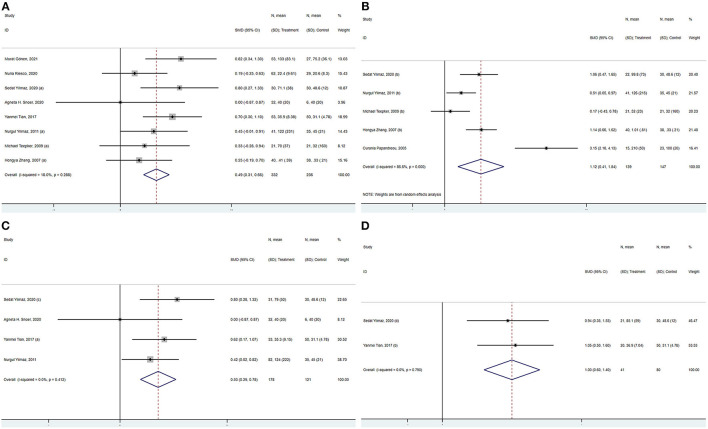
Forest plot for the association between S100B levels and migraine **(A)** in the interictal period, **(B)** in the ictal period, **(C)** without aura, and **(D)** with aura. SMD, standardized mean difference; CI, confidence interval.

The combined results of the meta-analysis (fixed-effects model, *I*^2^ = 18.0%, *P* = 0.288) indicated that S100B levels were significantly higher in interictal patients than in controls (SMD = 0.487, 95%CI: 0313–0.661, *P* < 0.001) (Figure 3A). Similarly, the combined results of the meta-analysis (random-effects model) comparing the S100B levels of migraine patients in the ictal period to that of controls suggested that SMD was 1.123 (95%CI: 0.409–1.836, *P* = 0.002), the corresponding *I*^2^-statistic was 86.6% (*P* < 0.001) (Figure 3B). The S100B levels of patients were not significantly difference in interictal period than ictal period (SMD = 0.240, 95%CI: −0.378 to 0.857, *P* = 0.447, *I*2 = 82.9%, *P*h = 0.001).

#### S100B Levels of Migraineurs Without and With Aura

S100B levels were significantly higher in migraineurs without aura than in controls in the meta-analysis pooling four studies (SMD = 0.534, 95%CI: 0.286–0.783, *P* < 0.001), the corresponding *I*^2^-statistic was 0.0% (*P* = 0.412) (Figure 3C). S100B levels were significantly higher in migraine patients with aura than in controls (SMD = 0.999, 95%CI: 0.598–1.400, *P* < 0.001, *I*^2^ = 0.0%, *P* = 0.790) (Figure 3D). Migraine patients without aura in the interictal periods also had significantly increased S100B levels in peripheral blood compared with healthy controls (SMD = 0.474, 95%CI: 0.173–0.774, *P* < 0.001, *I*^2^ = 0.0%, *P* = 0.460). The S100B levels were not significantly difference in patients without aura than with aura (SMD = 0.146, 95%CI: −0.247 to 0.539, *P* = 0.466, *I*2 = 0.0%, *P*h = 0.860).

#### S100B Levels of Patients With Chronic Migraine and Episodic Migraine

S100B levels were not significantly difference in patients with chronic migraine than in controls in the meta-analysis pooling two studies (SMD = 0.563, 95%CI: −0.086 to 1.212, *P* = 0.089), the corresponding *I*^2^-statistic was 68.1% (*P* = 0.076). S100B levels were also not significantly difference in patients with episodic migraine than in controls (SMD = 0.527, 95%CI: −0.369 to 1.423, *P* = 0.249, *I*^2^ = 79.4%, *P* = 0.028).

### Results of Subgroup Analysis

The overall estimation of S100B in migraine showed significant heterogeneity. We performed subgroup analyses to further elucidate the association between S100B and migraine, and to explore the source of heterogeneity. Subgroup analyses by age group were also performed. Pooled data demonstrated that more significant differences in blood S100B levels between migraine patients and healthy controls were found in children (SMD = 3.15, 95% CI: 2.18–4.13) than in adults (SMD = 0.63, 95% CI: 0.44–0.82) and other age groups (including both minors and adults, and unknown age group) (SMD = 0.32, 95% CI: 0.02–0.62). The heterogeneity decreased notably after the age subgroup analysis, which indicated that age or only one study on children may be an important source of heterogeneity. In addition, the results of stratification by country, diagnosis of migraine, and assay method of S100B indicated a correlation between elevated S100B and migraine (Table 4). The I2 was reduced to below 50% in China, International Classification of Headache Disorders, 3/2nd edition (ICHD-III/II), enzyme-linked immunosorbent assay (ELISA), and electrochemical luminescence immunoassay (ECLIA) subgroups, which shows that heterogeneity may be related to the assessment of migraine and assay method of S100B.

**Table 4:** Results of subgroup analyses.

**Subgroup**	***N***	**Random-effects model**		**Fixed-effects model**		**Heterogeneity**	
		**SMD (95% CI)**	***P***	**SMD (95% CI)**	***P***	***I*** **^2^**	**Ph**
**1: Age**							
Children	1	3.15 (2.18, 4.13)	<0.001	3.15 (2.18, 4.13)	<0.001	/	/
Adults	6	0.63 (0.44, 0.82)	<0.001	0.63 (0.44, 0.82)	<0.001	0.0%	0.479
Others	2	0.32 (0.02, 0.62)	0.034	0.32 (0.02, 0.62)	0.034	0.0%	0.448
**2: Country**							
China	2	0.67 (0.39, 0.95)	<0.001	0.67 (0.39, 0.95)	<0.001	0.0%	0.838
Europe	7	0.72 (0.23, 1.21)	0.004	0.57 (0.38, 0.77)	<0.001	82.6%	<0.001
**3: Migraine assessment**							
ICHD-III/II	5	0.56 (0.28, 0.84)	<0.001	0.58 (0.36, 0.79)	<0.001	38.9%	0.162
IHS	4	1.00 (0.22, 1.79)	0.012	0.65 (0.41, 0.89)	<0.001	89.3%	<0.001
**4: Assay method of S100B**							
ELISA	4	0.59 (0.33, 0.85)	<0.001	0.59 (0.38, 0.81)	<0.001	31.0%	0.226
ECLIA	3	0.50 (0.15, 0.84)	0.005	0.51 (0.22, 0.79)	0.001	23.7%	0.270
Other	2	1.70 (1.09, 4.49)	0.233	0.95 (0.48, 1.41)	<0.001	96.1%	<0.001

*ICHD-III, International Classification of Headache Disorders, 3rd edition; ICHD-II, International Classification of Headache Disorders, 2nd edition; IHS, criteria of the International Headache Society; ELISA, enzyme-linked immunosorbent assay; ECLIA, electrochemical luminescence immunoassay*.

### Sensitivity Analysis

**Figure 4:**
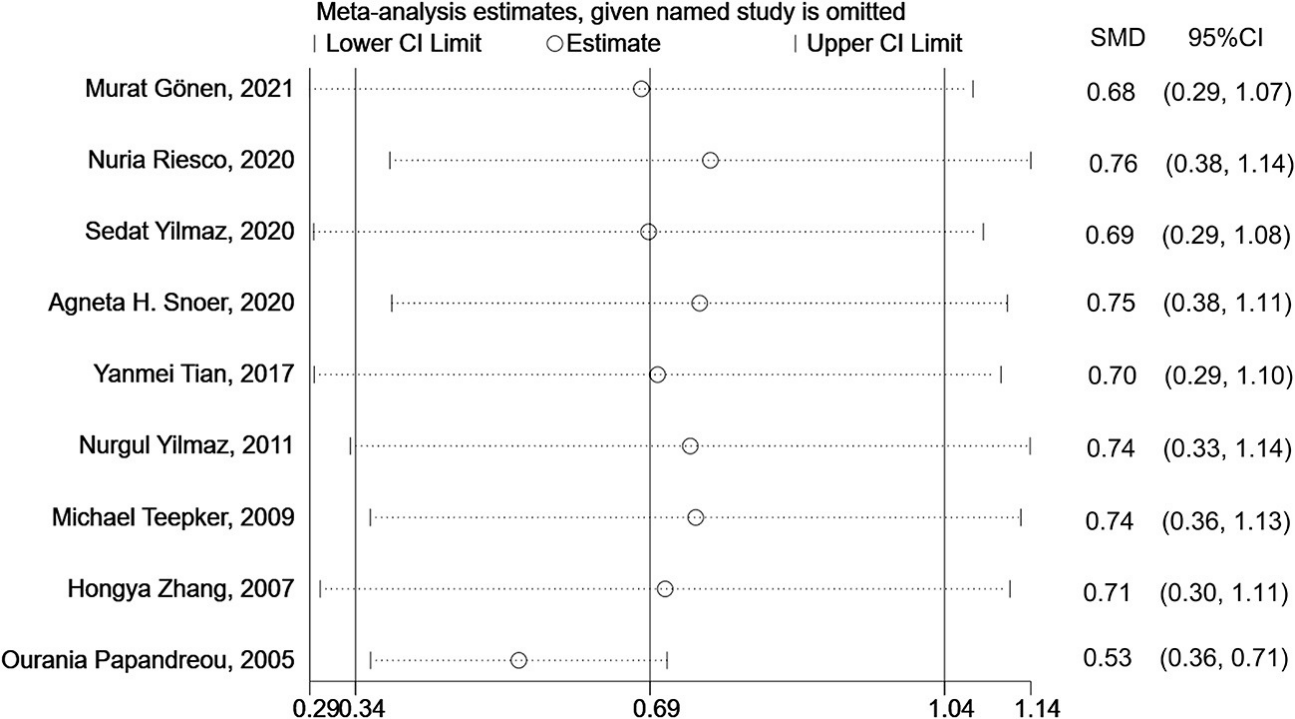
Sensitivity analysis for the association between S100B levels and overall migraine. SMD, standardized mean difference; CI, confidence interval.

Sensitivity analysis (including a minimum of three studies) was performed to assess and evaluate the stability and reliability of the overall SMD by leave-one-out cross-validation, which repeated the analysis after the sequential exclusion of each study. The results were not substantially altered after the serial exclusion of individual studies (Figure 4 and Supplementary Figure). Thus, these strategies revealed that the results were stable.

### Publication Bias

Since the number of studies we included was <10, the results of the Begg rank correlation test (*P* > |z| = 0.754) and Egger linear regression (*P* = 0.220) were only used to roughly evaluate publication bias. Positive results may be more likely to be published, which may lead to publication bias in this study.

## Discussion

In recent years, some studies have explored the S100B level in migraine patients, but the sample size of participants was small, and the results were not consistent. Our study aimed to review existing studies to synthesize data and uncover further evidence to confirm the correlation between S100B levels and migraine. To the best of our knowledge, this is the first meta-analysis to investigate the correlation between migraine and S100B. The findings of the current meta-analysis including nine studies showed that S100B levels were indeed significantly increased in migraineurs than in the healthy control group, although the results of previous clinical studies on this topic were not consistent.

In 2005, Papandreou et al. first reported significantly elevated S100B levels in pediatric patients with migraine during the ictal period (23). This may indicate that brain cell damage caused by local inflammation leads to the elevation of S100B levels. Subsequently, several studies found that the S100B level in migraine during the interictal period also increased (22, 24, 26–30). These findings suggest that migraine may be an ongoing or progressive disorder with a subclinical course, rather than a paroxysmal disorder characterized by intermittent headaches. During this interval, there are persistent pathological changes and processes in the nervous system. Evidence is increasing for other functional and structural brain changes that occur over time in individuals with migraine (31–33). However, some studies hold different opinions, making the correlation between S100B and migraine controversial. Therefore, a meta-analysis is needed to further verify these results. Our study included nine studies, of which eight contained S100B data of migraineurs during the interictal phase, five comprised ictal data, four had S100B data of migraineurs without aura, two had S100B data of migraineurs with aura, and two had S100B data of patients with episodic migraine and chronic migraine. S100B levels increased in many types of migraine, except chronic migraine and episodic migraine. This may imply that S100B is involved in the pathological process of the occurrence and development of migraine. In these two studies including data of patients with episodic migraine and chronic migraine, the S100B levels of both chronic and episodic migraine in the Riesco et al. study were not significant increases from those in the control group (24), unlike the findings of Gönen et al. (22). This may be related to the fact that preventive medication was not withdrawn before blood samples were collected in the Riesco et al. study. Preventive medication may ameliorate the pathological process of migraine and reduce the expression of S100B at the same time. Due to the small number of included articles, this finding also affected the results of the meta-analysis of the S100B levels of patients with chronic migraine and episodic migraine.

Subgroup analyses were also conducted, and we found that S100B increased more significantly in the children group after stratification by age group. Within the different age subgroups, *I*^2^ was 0.0%. This implies that age may be an important source of heterogeneity. Some previous studies have shown that migraineurs experience changes in structure (34) and metabolism (35) in their brains as they age. Some studies have suggested that the expression of S100B increases in the cortex and hippocampus by activating astrocytes with advancing age. These results indicate that S100B may play an important role in brain development and the establishment of proper brain functions. Upregulation of S100B in aging may be relevant to the age-related pathological state of the brain (36). Age could be considered an important factor influencing both S100B expression and migraine progression. However, since there was only one study on children in the subgroup analysis, and the quality evaluation of this study was low, the above results may not be reliable. Therefore, in the future, age stratification is needed to explore the elevated S100B levels in patients with migraine.

S100B is an acidic protein that binds to zinc (Zn^2+^) and calcium (Ca^2+^) and exists in the nucleus and cytoplasm of a broad range of cells (37–39). Intracellular S100B modulates a series of biological activities, such as cell proliferation, survival, differentiation, regulation of calcium homeostasis, enzyme regulation, and interaction with the cytoskeleton (40). Extracellular S100B binds to the receptor for advanced glycation end products (RAGE), which is a cell surface receptor of immunoglobulin present on various cell types (41, 42), initiates a complex intracellular signaling cascade (43), up-regulates nitric oxide synthase (NOS), inducing the release of NO and the production of reactive oxygen species (ROS) and NO-dependent neuronal and glial death (44, 45), facilitating glutamate-mediated death of neurons and upregulation of COX-II expression in microglia (46). Elevated S100B has been observed in patients with many neurological diseases, such as brain injury (47), Parkinson's disease (48), Alzheimer's disease (49), epilepsy (50), and multiple sclerosis (MS) (51). Elevated S100B was also observed to participate in the subsequent signaling pathway and inflammation in many animal models of neurological diseases.

Previous research has shown that extracellular signal-regulated kinases (ERK) are modulated by extracellular S100B protein in astrocyte cultures, resulting in increased activation of phosphorylation products (52). ERK and p38 activated by inflammatory factors participate in the generation and maintenance of inflammatory nociceptive hypersensitivity (53). There is little experimental research on the association between S100B levels and migraine. Chemical stimulation has been found to increase the expression of S100B and p38 in the trigeminal nerves in rat models of capsaicin-induced migraine (19, 20). A significant increase in p-ERK protein and S100B expression was also observed in the trigeminal ganglia of rat models of nitroglycerin (NTG) -induced migraine (20). Therefore, glial S100B expression and subsequent upregulation of the p-ERK and p38 pathways may be involved in trigeminal nociceptive hypersensitivity and the pathological process of migraine.

In some neurological diseases, some measures also play a role in ameliorating diseases and reducing the expression of S100B at the same time. S100B knockout mice showed reduced Aβ plaque formation in the cortical region in an experimental Alzheimer's disease model (54). In addition, arundic acid inhibits astrocytic S100B synthesis and induced the protection of dopaminergic neurons against 1-methyl-4-phenyl-1,2,3,6-tetrahydropyridine (MPTP) toxicity in a mouse model of Parkinson's disease (55). Similarly, arundic acidinhibits both astrocytic over-expression of S100B and the subsequent activation of signaling pathways in the peri-infarct area, which may be shared by ischemic stroke or even multiple sclerosis (56). In a rat model of epilepsy, resveratrol, metformin, dexamethasone, as anti-inflammatory and neuroprotective agents, also significantly reduced the levels of S100B participating in neuroinflammatory processes accompanying epilepsy (57–59). The above studies show that S100B is indeed involved in the process of many neurological diseases. In animal experiments on migraine, tetrandrine can suppress NTG-induced overexpression of S100B and p-ERK in satellite glial cells of the trigeminal ganglia and significantly alleviate trigeminal pain sensitization. This result suggests that S100B may play an important pathological role in the occurrence and maintenance of migraine. The study of S100B level in migraine may provide more directions for the study of migraine pathological mechanisms.

### Strengths and Limitation

This study has strengths and limitations. Its strengths lie in this is the first meta-analysis on this topic, and we investigated the relationship between various types of migraine and the level of S100B. These may contribute to the study of the pathological mechanism of migraine.

There are also some limitations in the present study that cannot be neglected. The present research may be biased due to the inability to obtain unpublished literature and the inclusion of only Chinese and English literature. Although this is the first meta-analysis on this topic, the quality of the research results may be affected by the scarcity of related studies and the small sample size of the included studies. Large-scale studies are needed to provide more deterministic evidence. Although we have found an important source of the overall heterogeneity through age subgroups, there was only one article in the children subgroup, and more studies in this age group are needed in the future to confirm the relationship between S100B and child migraine. Due to the lack of more detailed characteristics of migraine, such as the disease duration and frequency of attacks in the included studies, our research was not been able to further explore the changes of S100B in migraines of different severity, which requires further research.

## Conclusion

In summary, this study provides evidence that S100B levels were significantly increased in migraine patients compared to controls, and helps to understand the pathological mechanism of migraine and the possible implementation of S100B-targeted therapies for migraine.

## Data Availability Statement

The original contributions presented in the study are included in the article/Supplementary Material, further inquiries can be directed to the corresponding author/s.

## Author Contributions

WL guided the design of the study and revised the manuscript. CC designed the study, screened the literature, extracted data, performed the statistical analysis, and wrote the manuscript. RZ and MC supported the screening of literatures and extracted data. NL supported the visualization of the results. All authors contributed to the article and approved the submitted version.

## Conflict of Interest

The authors declare that the research was conducted in the absence of any commercial or financial relationships that could be construed as a potential conflict of interest.

## Publisher's Note

All claims expressed in this article are solely those of the authors and do not necessarily represent those of their affiliated organizations, or those of the publisher, the editors and the reviewers. Any product that may be evaluated in this article, or claim that may be made by its manufacturer, is not guaranteed or endorsed by the publisher.
