# Supplementary figures and images for: Elevated Blood S100B Levels in Patients With Migraine: A Systematic Review and Meta-Analysis
Source: Front Neurol. 2022 Jul 14;13:914051. doi: 10.3389/fneur.2022.914051 (PMC9329586; doi:10.3389/fneur.2022.914051)

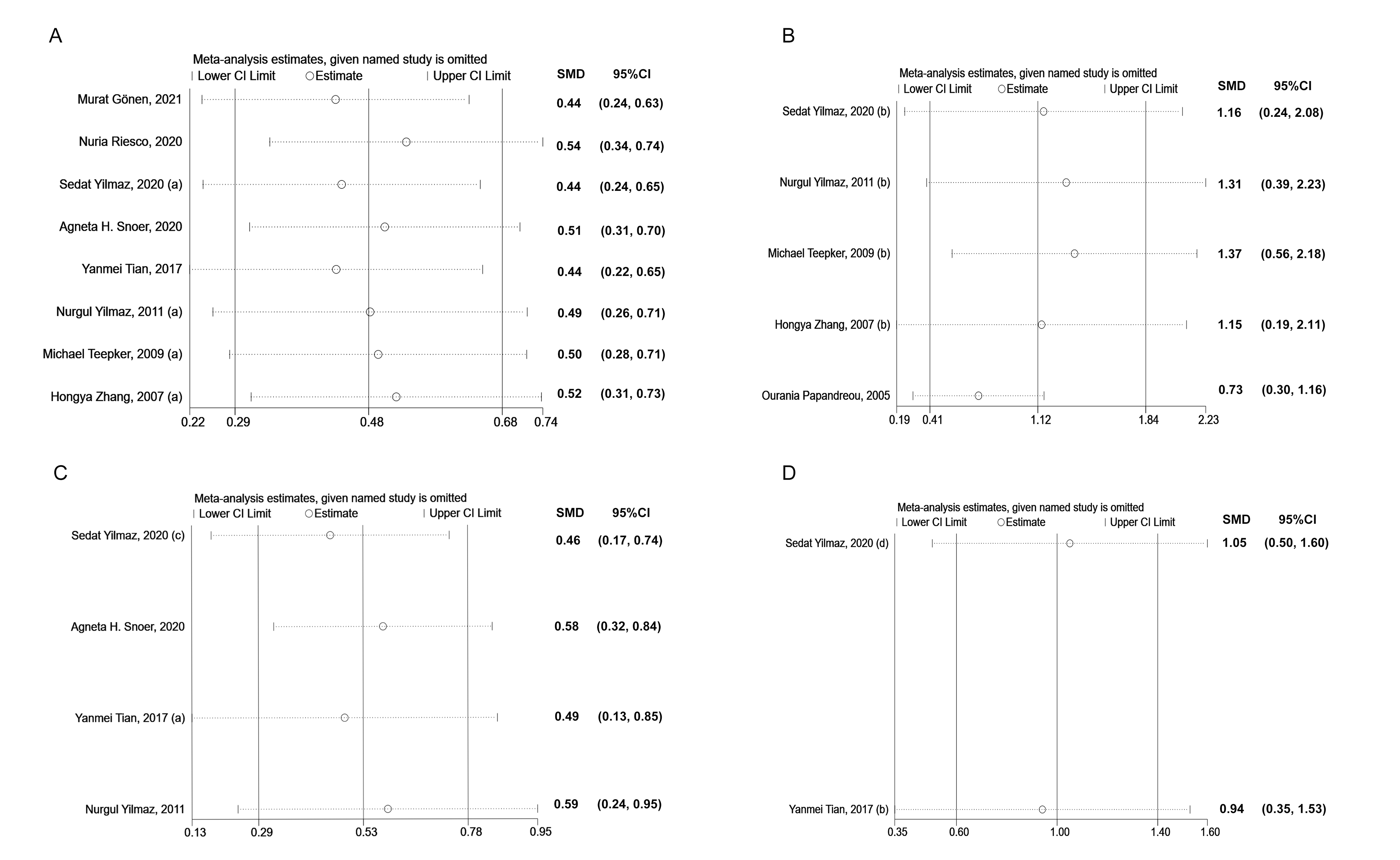

Supplement: Supplementary file 1 [file Figure_1.TIF]
